# Supplementary material for: Artificial nighttime lighting impacts visual ecology links between flowers, pollinators and predators
Source: Nat Commun. 2021 Jul 6;12:4163. doi: 10.1038/s41467-021-24394-0 (PMC8260664; doi:10.1038/s41467-021-24394-0)
Supplement: Supplementary file 1 — Supplementary Information [file 41467_2021_24394_MOESM1_ESM.pdf]

Supplementary information for 'Artificial nighttime lighting impacts visual ecology links between flowers, pollinators and predators'

Emmanuelle S. Briolat[1], Kevin J. Gaston[2], Jonathan Bennie[2], Emma J. Rosenfeld[2], Jolyon Troscianko[1]*

[1]Centre for Ecology & Conservation, University of Exeter, Penryn

[2]Environment & Sustainability Institute, University of Exeter, Penryn

*Corresponding author: jt@jolyon.co.uk

SUPPLEMENTARY FIGURES

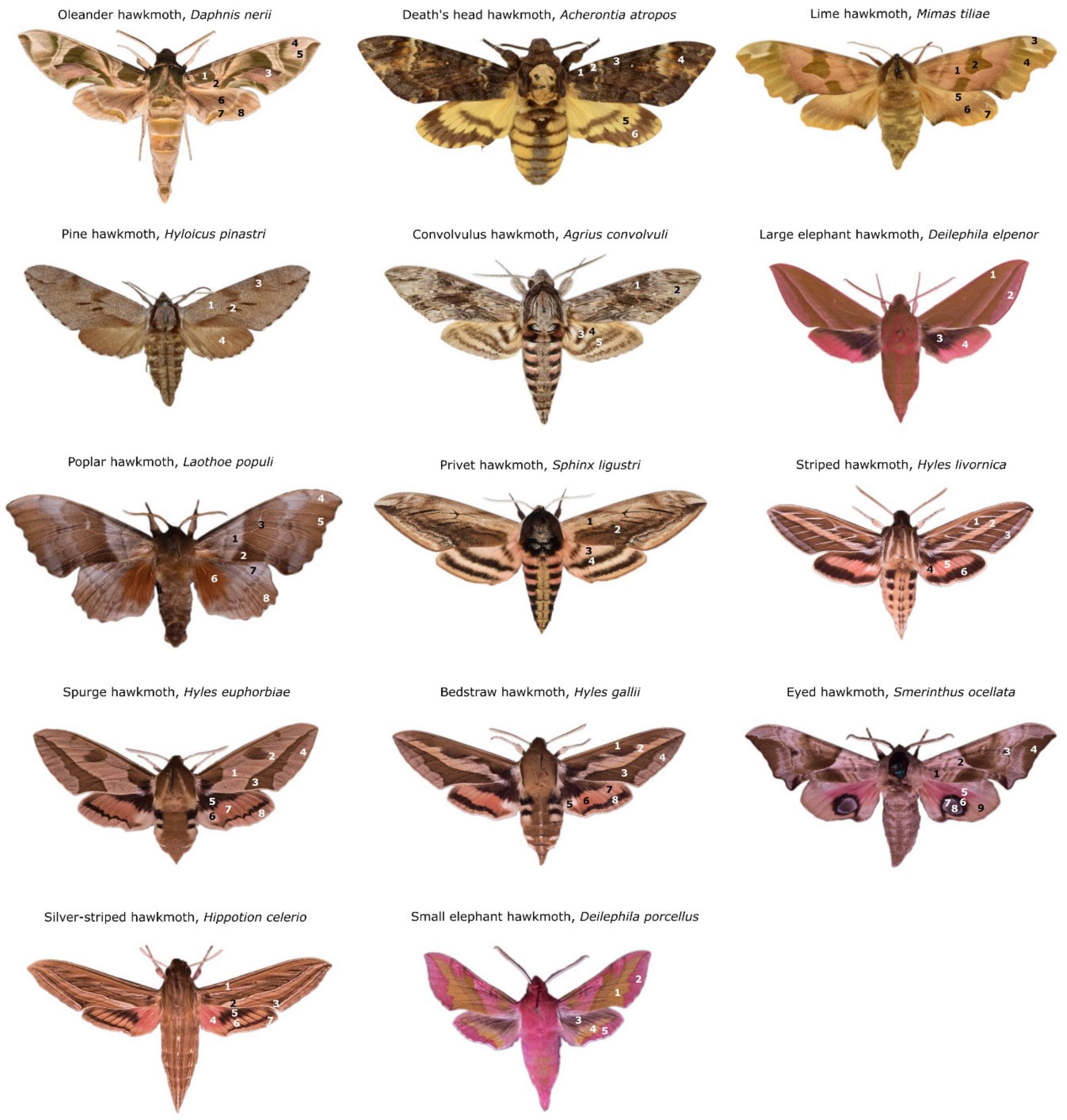

Supplementary Figure 1: Areas selected for colour measurements for each hawkmoth species.

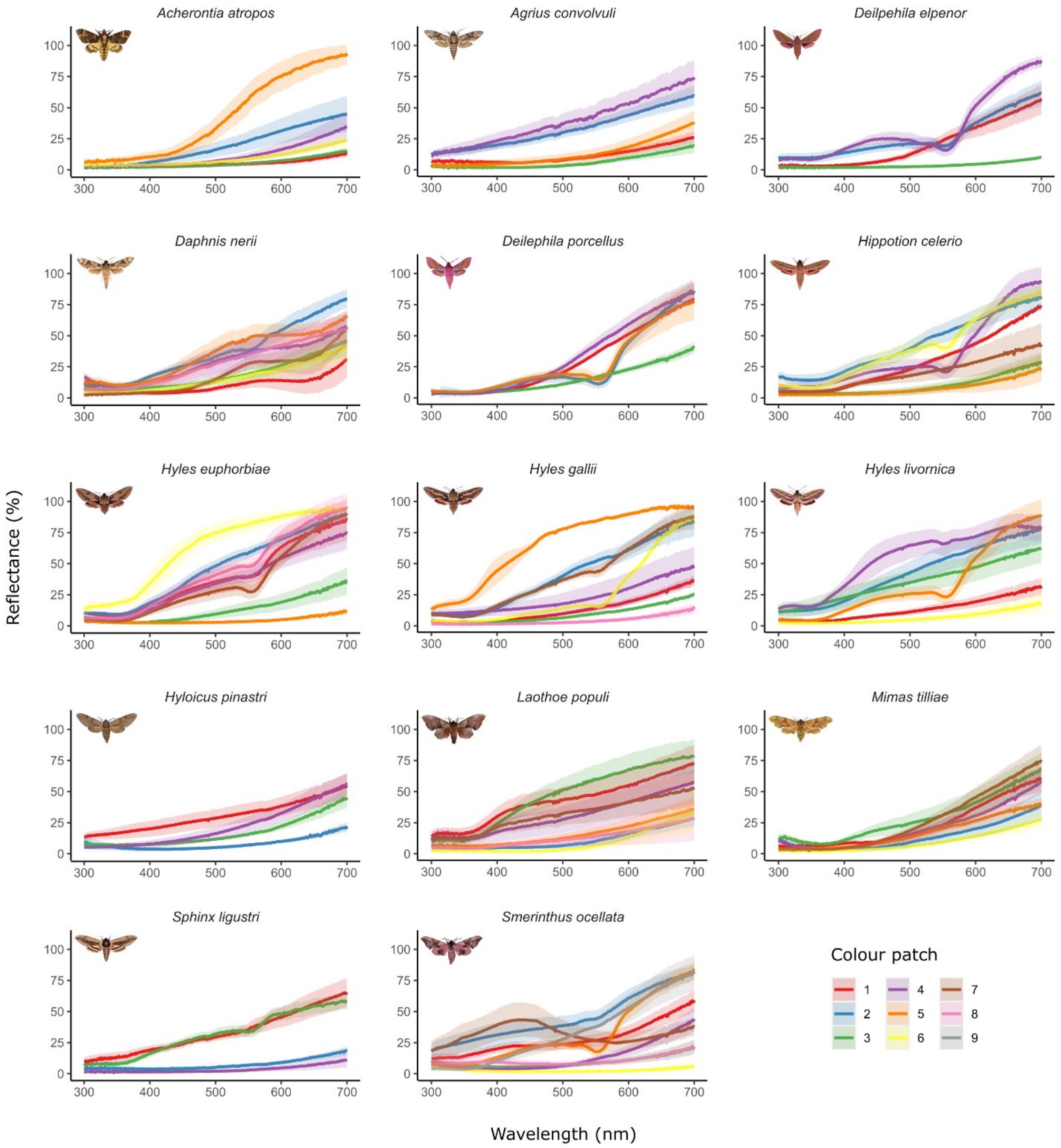

Supplementary Figure 2: Average reflectance spectra for the colour patches selected for each moth species, across 5 individuals per species. Patch numbers correspond to those shown on the hawkmoth specimens in Supplementary Fig. 1. Shading represents standard error.

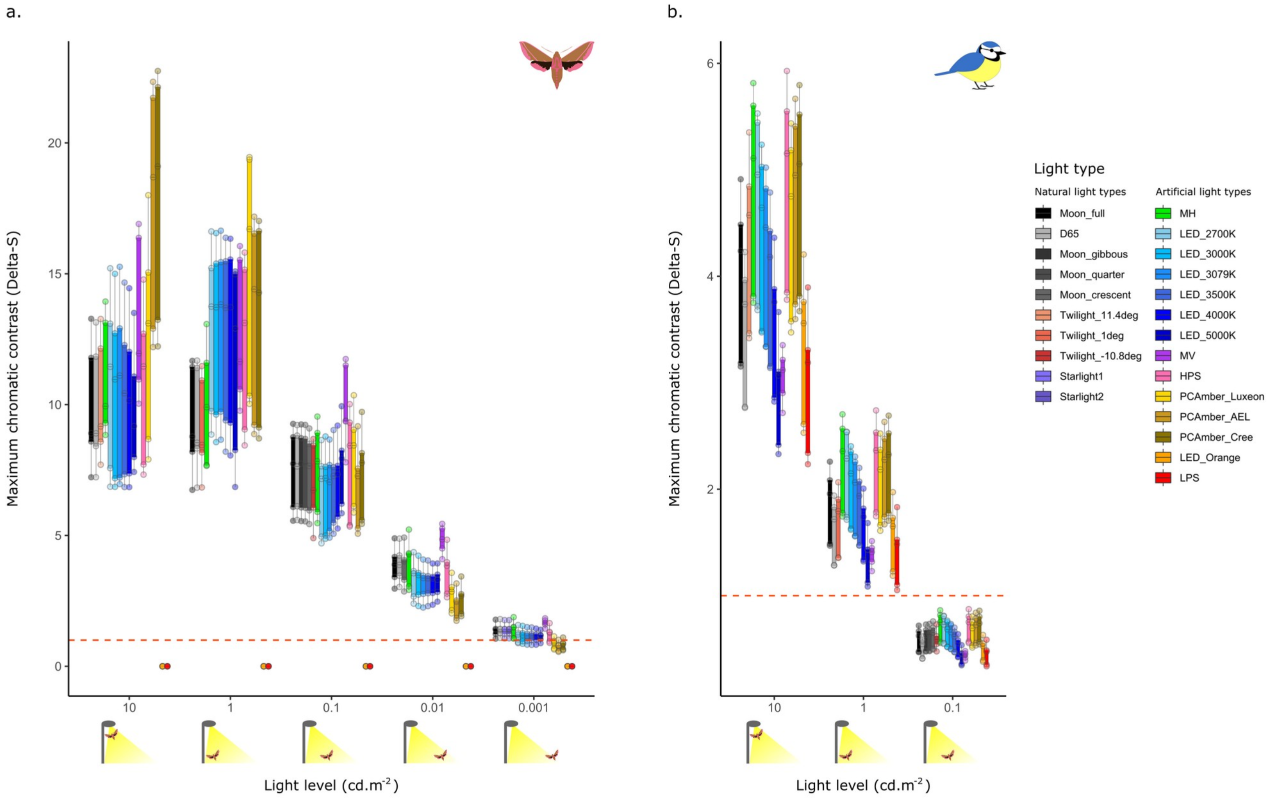

<u>Supplementary Figure 3:</u> Maximum chromatic contrast between wing colours in specimens of one hawkmoth species, *D. elpenor* (N=5), under different light types and light levels, as perceived by hawkmoths (a) and blue tits (b). Data are represented as boxplots where the middle line is the median, the lower and upper hinges correspond to the first and third quartiles, the upper whisker extends to the maximum value, and the lower whisker extends to the minimum value. The dashed line represents a putative threshold for colour discrimination (Delta-S=1). Hawkmoths can see colour at substantially lower light levels than typical passerines, resulting in higher chromatic contrasts for hawkmoths.

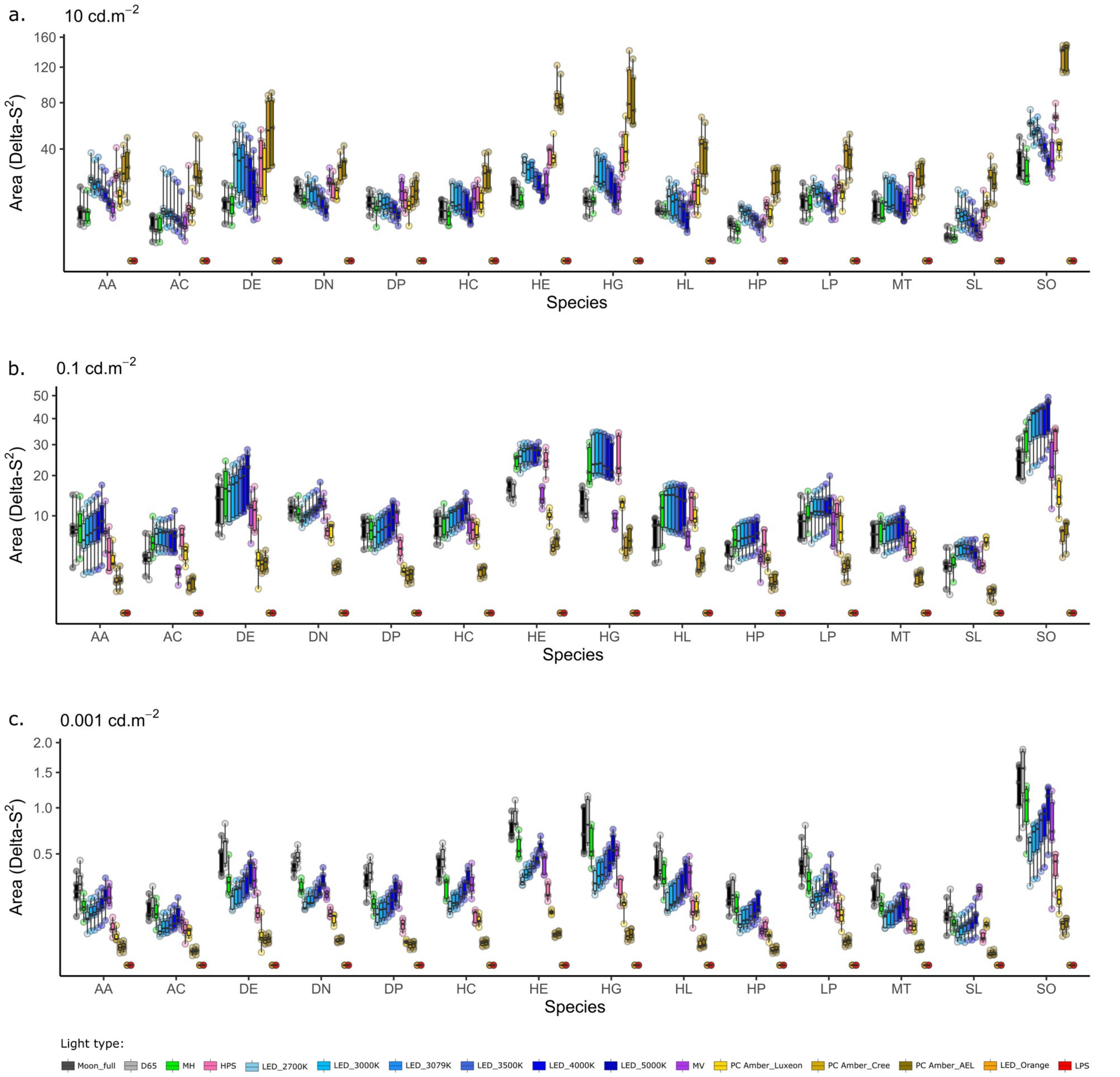

Supplementary Figure 4: Areas occupied by the colours of each specimen (N=5 per species) in the hawkmoth RNL chromaticity space, plotted by species and light type, for 3 light levels: (a) 10 cd.m^{-2}, (b) 0.1 cd.m^{-2}, (c) 0.001 cd.m^{-2}). Data are represented as boxplots where the middle line is the median, the lower and upper hinges correspond to the first and third quartiles, the upper whisker extends to the maximum value, and the lower whisker extends to the minimum value.

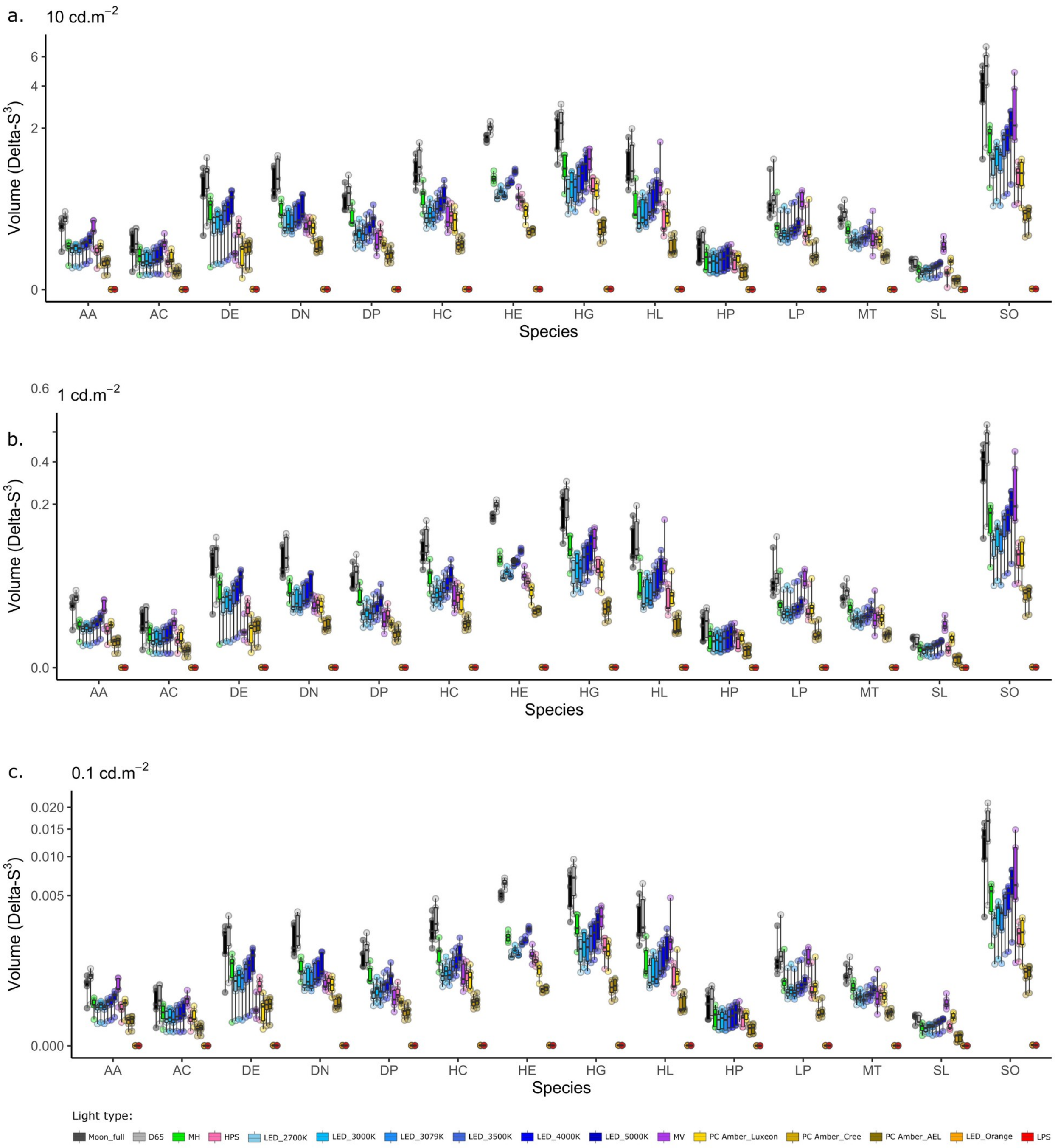

<u>Supplementary Figure 5:</u> Volumes occupied by the colours of each specimen (N=5 per species) in the blue tit RNL chromaticity space, plotted by species and light type, for 3 light levels: (a) 10 cd.m^{-2}, (b) 1 cd.m^{-2}, (c) 0.1 cd.m^{-2}). Data are represented as boxplots where the middle line is the median, the lower and upper hinges correspond to the first and third quartiles, the upper whisker extends to the maximum value, and the lower whisker extends to the minimum value.

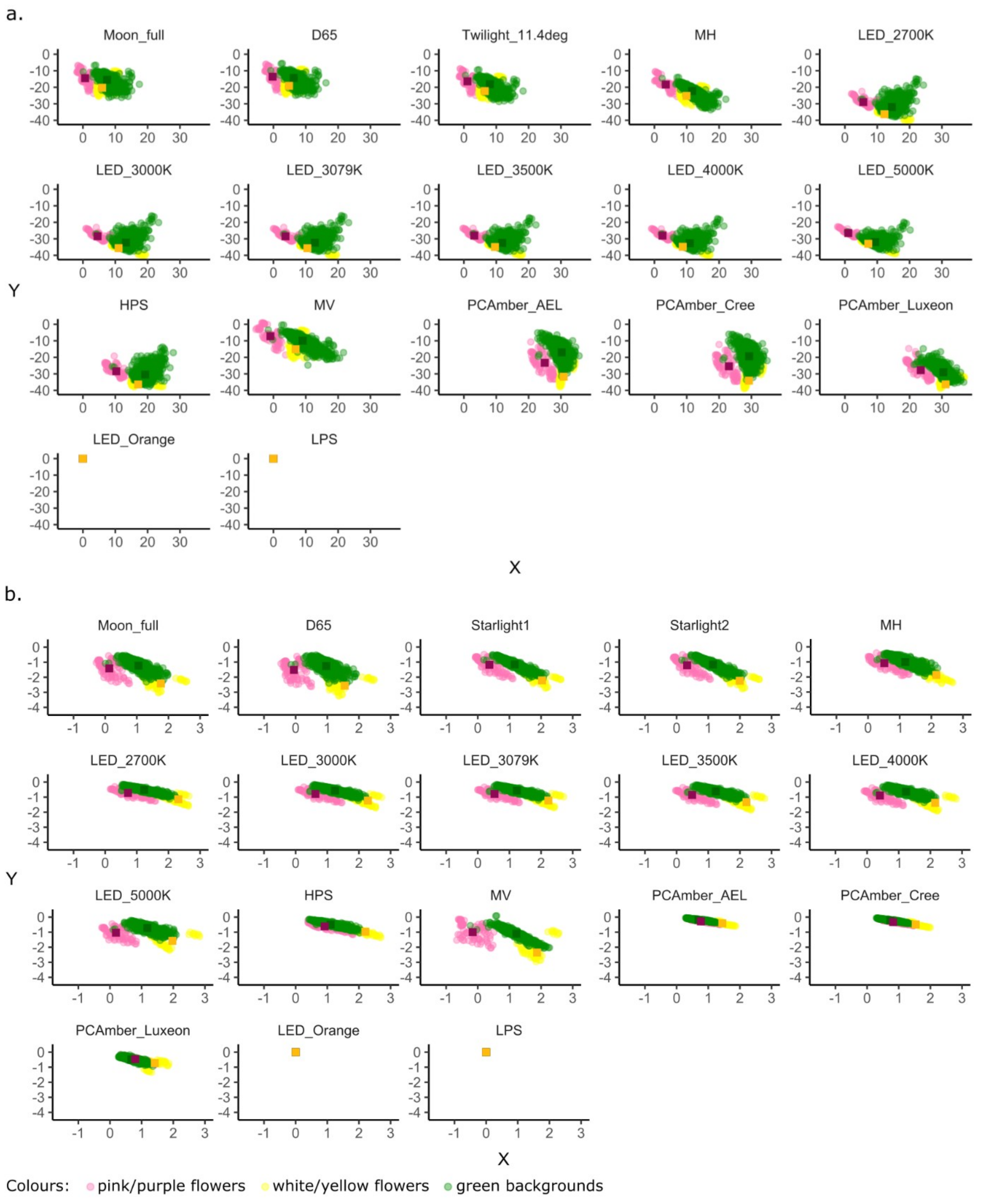

<u>Supplementary Figure 6:</u> Flower and green background colours plotted in the hawkmoth RNL space, for each light type and light levels corresponding to (a) 10 cd.m^{-2} and (b) 0.001 cd.m^{-2}. Darker squares represent the geometric means of each group.

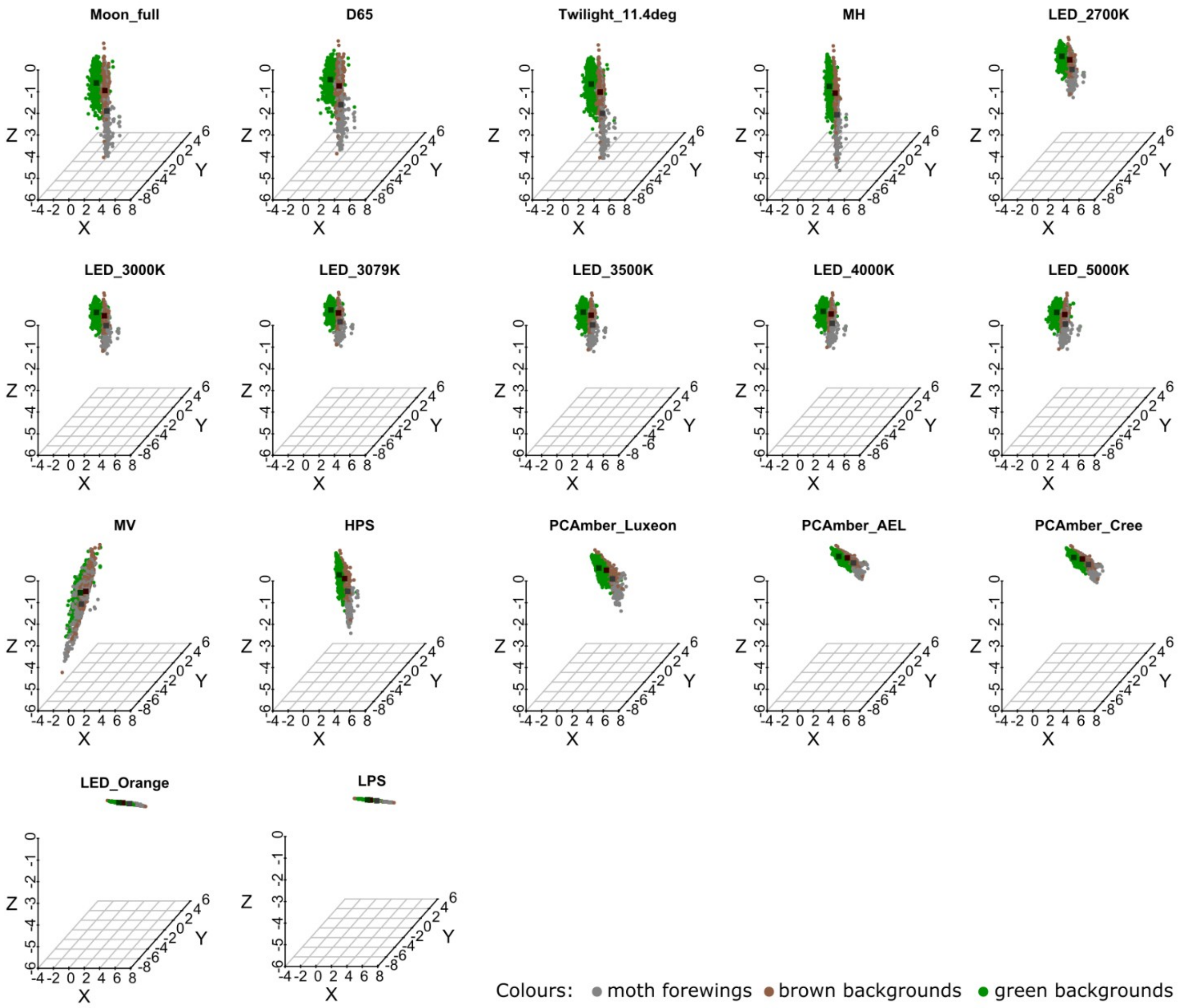

Supplementary Figure 7: Moth forewing and natural background colours plotted in the blue tit RNL space, for each light type, for light levels corresponding to 10 cd.m^{-2}. Darker squares represent the geometric means of each group.

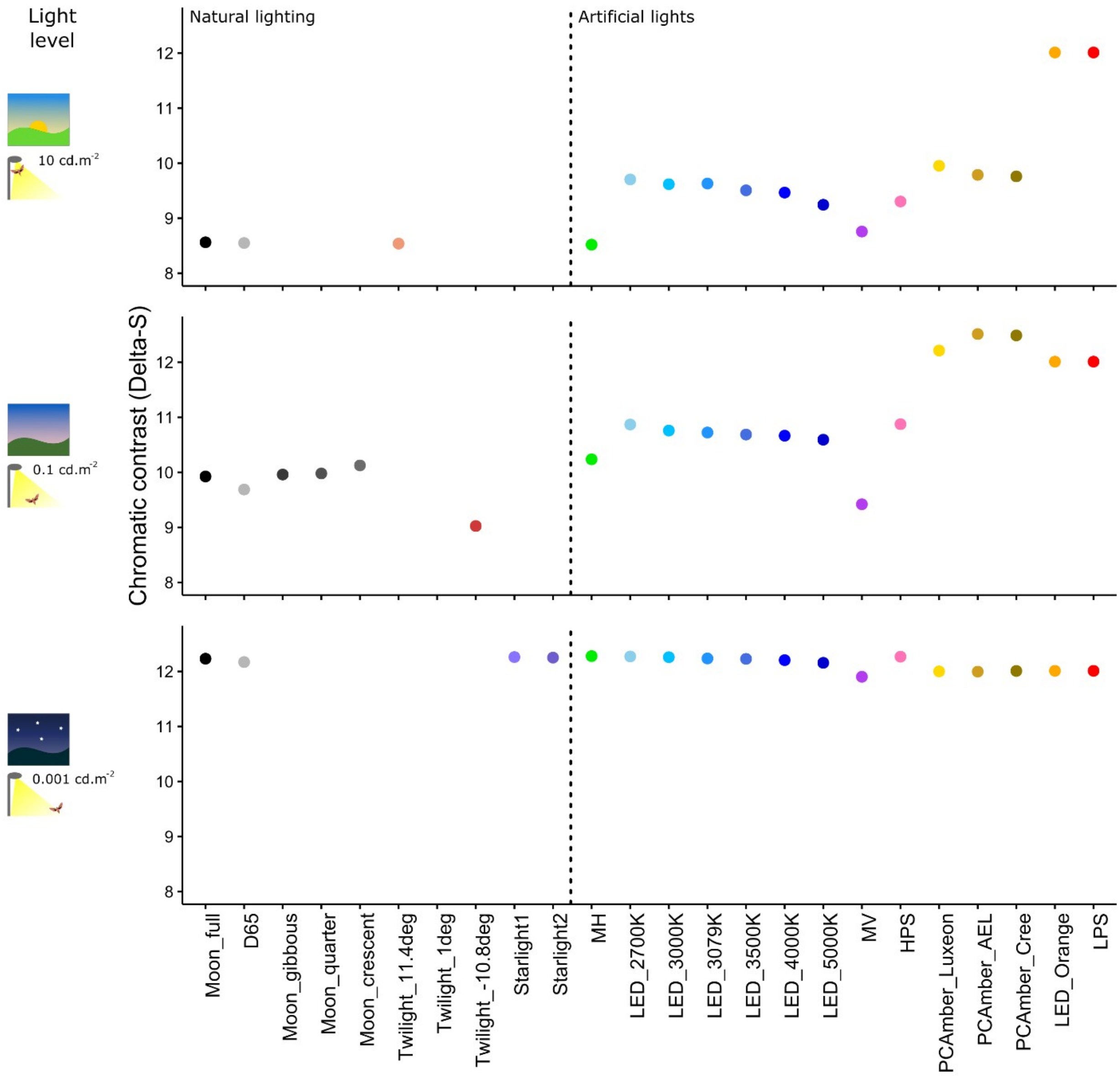

Supplementary Figure 8: Chromatic contrasts between moth forewing and natural background colours as perceived by the blue tit visual system in daylight, for pairs of colours selected to have a perfect colour match to hawkmoth vision under different light regimes at night. Mean and 95% confidence intervals for chromatic contrasts, modelled for hawkmoths at three light levels.

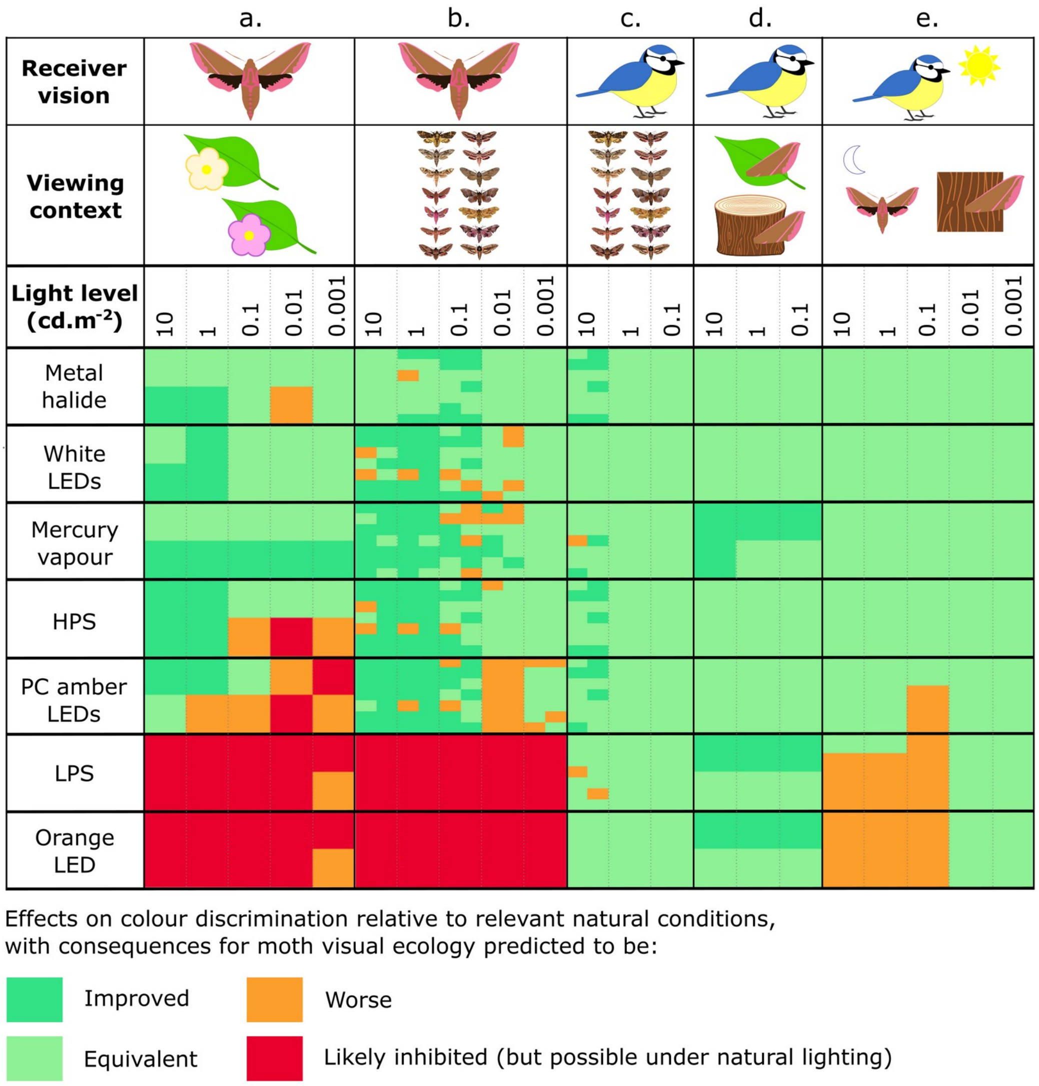

Supplementary Figure 9: Effects of artificial lighting on colour perception, compared to natural lighting conditions at equivalent light levels. Lights are labelled as performing better (green) or worse (orange/red) than a relevant natural illuminant at each light level, from the hawkmoth perspective. Reference illuminants are dusk (sun 11.4° above the horizon), twilight (sun 1° above the horizon), full moon, crescent moon and starlight (sunspot maximum) for light levels from 10 to 0.001 $cd.m^{-2}$ respectively. Hawkmoth perception was modelled for: a) contrast of white/yellow flowers (top of cells), and pink/purple flowers (bottom of cells) against natural foliage backgrounds; and b) maximum internal contrast for intraspecific signals (fore- and hindwings, 14 species positioned alphabetically in each cell). Blue tit perception was modelled for: c) hawkmoth internal contrast (signalling, fore- and hindwing); d) hawkmoth forewing camouflage against green (top of cells) or brown (bottom of cells) natural backgrounds; and e) hawkmoth background selection under artificial light followed by blue tit detection under diurnal predatory search behaviour. Thresholds for a) and d) are set where there is no overlap between confidence intervals compared to the reference natural light type. Thresholds in b) and c) are determined by the combined estimated

effect of light type and the light:species interaction in models of the impact of lighting on maximum chromatic contrasts, with thresholds for differences met when the absolute value of this estimate is greater than 1 Delta-S. Thresholds for e) are based on statistical differences against the reference illuminant representing colour mismatches >1 Delta-S. For white and PC amber LEDs, the statistical results in b, c and e are based on an LED in the middle of our CCT range (CCT=3079K) and PC amber Cree respectively, so conclusions may not apply to white LEDs with extreme CCT values.

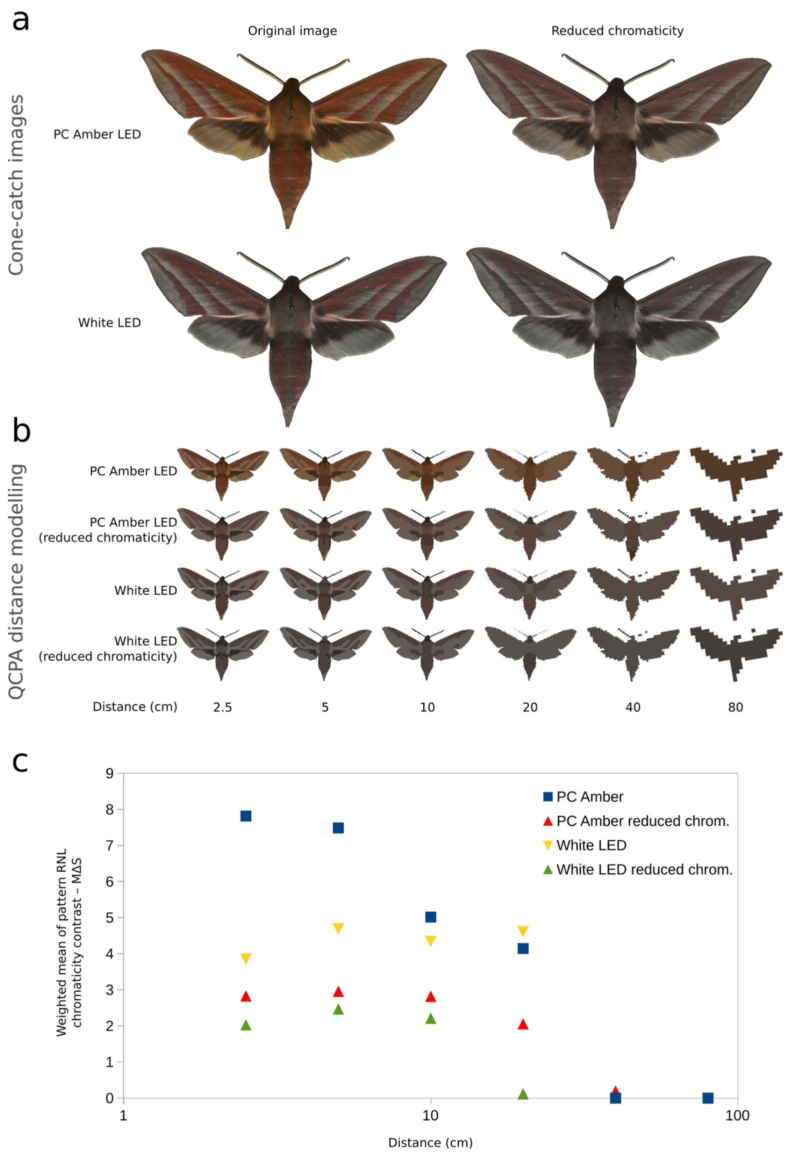

Supplementary Figure 10: Spatiochromatic modelling of suprathreshold chromatic differences. The RNL model is not designed to accurately estimate suprathreshold chromatic distances, however, any adjacent colours will effectively blend together at a certain viewing distance for any viewer, creating a spatiochromatic interaction between viewing distance and chromatic discrimination. This figure demonstrates this effect in an elephant hawkmoth (*D. elpenor*). a) shows the original photographs mapped (using the micaToolbox,[14]) to blue tit cone-catch under PC amber and white LED illuminants. The right-hand images have had the chromaticity (distance of each pixel's colour to the achromatic point) halved. These images were then passed through the QCPA framework to model the images at different viewing distances to a blue tit (b). c) shows that internal wing contrast (weighted mean of chromaticity contrast, MΔS) will affect the maximum detection distance – the original images have maximum detection distances (MΔS > 3) of 20cm, while identical images with artificially reduced internal contrast reduces the maximum detection distance to 10cm. While this example focuses on internal wing contrasts, the same effects will be true for flower detection distances and forewing-background detection distances.

SUPPLEMENTARY TABLES

Supplementary Table 1: Illuminant spectra modelled.

Light type & spectral power distribution		Source
Moonlight	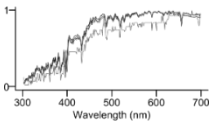	Data from Johnsen et al.[1]:<br>Spectra from full, gibbous, quarter and crescent moon phases; full moon 98% full, elevation 69°
D65 daylight	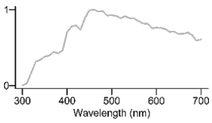	CIE D65 spectrum
Twilight	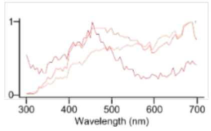	Data from Johnsen et al.[1]:<br>Sun at 11.4°, 1° and -10.8° above the horizon
Starlight	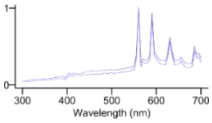	Data from Johnsen et al.[1]:<br>Sunspot maximum (starlight1) and sunspot minimum (starlight2)
Metal halide	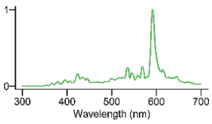	Measured under streetlights in Cornwall, UK[†‡]
High-pressure sodium	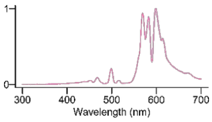	Measured under streetlights in Cornwall, UK[†‡]
Low-pressure sodium	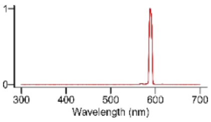	Measured under streetlights in Cornwall, UK[†‡]
Mercury vapor	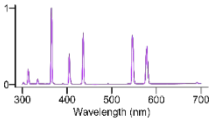	Measured for this study*[§]
White LEDs	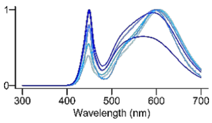	Philips Lumileds LUXEON RebelPlus spectra [‖]; Streetlight LED from Swanpool, Falmouth, UK (3079K) measured for this study*[§]
PC amber LEDs	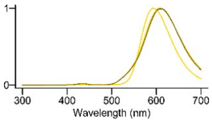	Philips Lumileds LUXEON Rebel*[†]; Cree PC amber & AEL 75W from Longcore et al.[15] (data available at: https://github.com/herf/ecological)
Orange LED	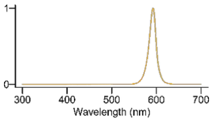	OSRAM.Tester.Orange from Elvidge et al.[16] (data available at: https://ngdc.noaa.gov/eog/night_sat/spectra.html)

[*] Measured using a Specbos 1211 spectroradiometer (JETI, Jena, Germany)

[†] Measured as radiance

[‡] Measured using a MAYA2000PRO spectrophotometer (Ocean Optic, Dunedin FL, USA)

[§] Measured as reflected radiance from a 99% Spectralon standard

‖ Data extracted from manufacturer's graphs using WebPlotDigitizer[12]

Supplementary Table 2: Provenance of hawkmoth specimens.

Species	Provenance	Date eclosed/collected	Location collected	Collector information
Acherontia atropos	Exeter RAMM	12/10/1993	Instow, Devon, UK	K. M. Hinchliff
	Exeter RAMM	06/06/1932	Exeter, Devon, UK	NA
	Exeter RAMM	October 1924	Rougemont grounds, Exeter, Devon, UK	A. Browne
	Exeter RAMM	19/11/1938	Topsham, Devon, UK	T. B. Pyne
	Exeter RAMM	1907	NA	Hellins collection
Agrius convolvuli	Exeter RAMM	27/08/1922	Lapford, North Devon, UK	Gay collection
	Exeter RAMM	October 1922	Exeter, Devon, UK	NA
	Exeter RAMM	1946	Braunton Burrows, North Devon, UK	Dr Wright
	Exeter RAMM	1946	Braunton Burrows, North Devon, UK	Dr Wright
	Exeter RAMM	19/09/1950	Woodbury, nr. Exeter, Devon, UK	NA
Daphnis nerii	Bristol Museum	August 1970	Persia	K. H. Poole collection, ECMAG1557
	Bristol Museum	August 1970	Persia	K. H. Poole collection, ECMAG1557
	Bristol Museum	17/06/1995	Goonhavern, Cornwall, UK	Frank Smith collection
	Exeter RAMM	June 1875	Ascot race course, Berkshire, UK	A. E. Studd, ref 28/6/73
	Exeter RAMM	4/7/1937	Karura forest, Kenya	Buckler collection
Deilephila elpenor	Bristol Museum	May 1975	Taunton, Somerset, UK	K. H. Poole collection, ECMAG1557
	Bristol Museum	25/05/1960	Weston-super-Mare, Somerset, UK	K. H. Poole collection, ECMAG1557
	Adrian Spalding	26/07/2002	Playing Place, Cornwall, UK	W. G. Tremewan
	Adrian Spalding	30/06/2002	Playing Place, Cornwall, UK	W. G. Tremewan
	Laura Kelley	25/07/2017	Cornwall, UK	Laura Kelley
Deilephila porcellus	Bristol Museum	10/06/1982	Picket Wood, Wiltshire, UK	G. E. Smith collection
	Bristol Museum	10/06/1982	Woodfalls, Wiltshire, UK	G. E. Smith collection
	Exeter RAMM	04/07/1933	Little Aston, Staffordshire, UK	F.H.Lees, ref 9/1997/56
	Exeter RAMM	04/07/1933	Little Aston, Staffordshire, UK	F.H.Lees, ref 9/1997/57

	Exeter RAMM	Jul-30	Little Aston, Staffordshire, UK	F.H.Lees, ref 9/1997/58
Hippotion celerio	Bristol Museum	10/09/1958	Bodinnick, S. E. Cornwall, UK	Frank Smith collection
	Bristol Museum	24/09/1913	Arundel, West Sussex, UK	Comey collection, specimen from Miss A. Middleditch
	Bristol Museum	1926-1936	Lebong Tandai, Benkoelen, Sumatra	Frank Smith collection
	Exeter RAMM	September 1869	Weston-super-Mare, Somerset, UK	G.F. Mathew, in Studd collection, ref 26/1942/39
	Exeter RAMM	August 1876	Ventnor, Isle of Wight, UK	E. Harris Jones, passed through collections of Stevens, Dr T. C. Melvill & Lees, ref 9/1997/51
Hyles euphorbiae	Bristol Museum	25/06/1965	NA	K. H. Poole collection, ECMAG1557
	Bristol Museum	06/08/1964	NA	K. H. Poole collection, ECMAG1557
	Bristol Museum	June 1990	Italy	K. H. Poole collection, ECMAG1557
	Bristol Museum	June 1990	Italy	K. H. Poole collection, ECMAG1557
	Exeter RAMM	13/3/1878	North Devon, UK	Studd collection
Hyles gallii	Bristol Museum	18/06/1979	Canary Islands	K. H. Poole collection, ECMAG1557
	Bristol Museum	13/06/1979	Canary Islands	K. H. Poole collection, ECMAG1557
	Bristol Museum	18/06/1979	Canary Islands	K. H. Poole collection, ECMAG1557
	Exeter RAMM	1916	North Cornwall, UK	L. W. Newman, in Lees collection
	Exeter RAMM	November 1914	North Cornwall, UK	G. B. Oliver, in Lees collection
Hyles livornica	Bristol Museum	August 1966	USA	K. H. Poole collection, ECMAG1557
	Bristol Museum	September 1985	Cornwall, UK	K. H. Poole collection, ECMAG1557
	Bristol Museum	26/5/2009	Trowbridge, Wiltshire, UK	G. E. Smith collection
	Exeter RAMM	18/8/2000	Sandy Acres, Broadclyst, South Devon, UK	S. Searle, ref 19/2018/72
	Exeter RAMM	18/8/2000	Sandy Acres, Broadclyst, South Devon, UK	S. Searle, ref 19/2018/74
Hyloicus pinastri	Exeter RAMM	08/06/1939	Suffolk, UK	Brooking Rowe collection
	Bristol Museum	22/06/1960	NA	from L. H. Newman, in K. H. Poole collection ECMAG 1557

	Bristol Museum	29/05/1959	NA	from L. H. Newman, in K. H. Poole collection ECMAG 1557
	Bristol Museum	04/07/1960	NA	from L. H. Newman, in K. H. Poole collection ECMAG 1557
	Bristol Museum	25/06/1960	NA	from L. H. Newman, in K. H. Poole collection ECMAG 1557
Laothoe populi	Exeter RAMM	03/08/1923	Lapford, North Devon, UK	Gay collection
	Adrian Spalding	08/07/2002	Playing Place, Cornwall, UK	W. G. Tremewan
	Adrian Spalding	13/07/2002	Playing Place, Cornwall, UK	W. G. Tremewan
	Adrian Spalding	04/08/2002	Playing Place, Cornwall, UK	W. G. Tremewan
	Bristol Museum	27/06/1960	Weston-super-Mare, Somerset, UK	K. H. Poole collection, ECMAG1557
Mimas tiliae	Exeter RAMM	June 1921	North Kent, UK	L. W. Newman, in the collections of F. Blanchford
	Exeter RAMM	17/05/1908	Exeter, Devon, UK	F. Blanchford
	Exeter RAMM	1917	Stoke Canon, Devon, UK	R. J. Ford
	Bristol Museum	18/06/1957	Perranporth, Cornwall, UK	Frank Smith collection
	Bristol Museum	03/06/1970	London, Greater London, UK	K. H. Poole collection, ECMAG1557
Smerinthus ocellata	Bristol Museum	22/06/1941	Sedbergh, Cumbria, UK	Frank Smith collection
	Bristol Museum	14/06/1945	Edgbaston, West Midlands, UK	Frank Smith collection
	Bristol Museum	12/06/1950	Bristol, UK	K. H. Poole collection, ECMAG1557
	Bristol Museum	03/06/1960	Weston-super-Mare, Somerset, UK	K. H. Poole collection, ECMAG1557
	Bristol Museum	28/05/1950	Bristol, UK	K. H. Poole collection, ECMAG1557
Sphinx ligustri	Exeter RAMM	1885	NA	N. P. Fenwick
	Exeter RAMM	04/07/1935	Instow, Devon, UK	K. M. Hinchliff
	Exeter RAMM	14/7/1895	Instow, Devon, UK	K. M. Hinchliff
	Adrian Spalding	02/07/2006	Playing Place, Cornwall, UK	W. G. Tremewan
	Adrian Spalding	06/07/2006	Playing Place, Cornwall, UK	W. G. Tremewan

Supplementary Table 3: Plant species sampled, with evidence of hawkmoth-mediated pollination or visits by adult hawkmoths.

Species	Collection locality	Evidence for hawkmoth pollination
Buddleia, *Buddleja* sp.	Penryn campus, Penryn, Cornwall, UK	Anecdotal photographic evidence of *H. gallii* feeding on buddleia flowers (https://www.alamy.com/stock-photo-bedstraw-hawkmoth-hyles-gallii-in-flight-feeding-on-buddleia-flowers-25859892.html)
Hedge bindweed, *Calystegia arvensis*	Penryn, Cornwall, UK	Related Convolvulaceae are pollinated by hawkmoths; hedge bindweed is reputed to be a hawkmoth-pollinated species, though direct evidence is lacking[17]; anecdotal evidence of *A. convolvuli* feeding on bindweed (http://filnorewoods.blogspot.com/2017/07/field-bindweed.html)
Knapweed, *Centaurea* sp.	Penryn campus, Penryn, Cornwall, UK	*Hemaris* spp. of hawkmoths in the USA feed on *Centaurea* knapweeds[18].
Red valerian, *Centranthus ruber*	Penryn, Cornwall, UK	*H. livornica* & *D. porcellus* recorded as feeding on these species in guides to UK moths[19,20].
Rosebay willowherb, *Chamerion angustifolium*	Penryn campus, Penryn, Cornwall, UK	*D. elpenor* adults recorded as visiting rosebay willowherb (http://plantlife.love-wildflowers.org.uk/wildflower_garden/grow_in_the_garden/rosebay_willowherb)
Foxglove, *Digitalis purpurea*	Penryn campus, Penryn, Cornwall, UK	Anecdotal photographic evidence of *D. elpenor*, *D. porcellus* & *Hyles lineata* (White-lined sphinx, from USA) on foxglove flowers (https://www.gettyimages.co.uk/detail/photo/stunning-elephant-hawk-moth-perching-on-a-foxglove-royalty-free-image/983176974; https://www.alamy.com/stock-photo-small-elephant-hawk-moth-deilephila-porcellus-adult-on-foxglove-powys-76524269.html; https://blog.mrsroadrunner.com/white-lined-sphinx-and-the-foxglove-flowers/)
Viper's bugloss, *Echium vulgare*	Penryn, Cornwall, UK	*D. porcellus* recorded as visiting viper's bugloss flowers in guide to UK moths[20].
Privet, *Ligustrum* sp.	Penryn, Cornwall, UK	Evidence of pollination by hawkmoths in Japanese privet species *L. ovalifolium*[21].
Common honeysuckle,	Penryn campus, Penryn, Cornwall,	Evidence of visits and pollen transport by *D. porcellus*[22,23]; *D. nerii*, *D.elpenor*, *D. porcellus* and *H. pinastri* recorded as visiting honeysuckle in guides to UK

Lonicera periclymenum	UK	moths[19,20].
Large-flowered evening primrose, *Oenothera glazioviana*	Constantine, Cornwall, UK	Evidence of hawkmoth visits to this genus in Japan[24].
Red campion, *Silene dioica*	Penryn campus, Penryn, Cornwall, UK	Observations of visits by *D. porcellus*[25]; Sphingidae reported as occasional pollinators of red campion[26].
White campion, *Silene latifolia*	Penryn campus, Penryn, Cornwall, UK	Observations of visits by *H. pinastri*[25]; Sphingidae reported as occasional pollinators of white campion[26].
Sea campion, *Silene uniflora*	Swanpool, Falmouth, Cornwall, UK	*D. porcellus* classed as a major pollinator of sea campion[26].
Verbena, *Verbena* sp.	Penryn campus, Penryn, Cornwall, UK	Related Verbenaceae are pollinated by hawkmoths, including *A. convolvuli* & *H. celerion*[27]; anecdotal photographic evidence of *H. lineata* visiting verbena flowers in the USA (https://www.oregonlive.com/hg/index.ssf/2016/08/grow_night-blooming_flowers_to.html)

SUPPLEMENTARY METHODS

Calculating quantal catch

Light source emission spectra were measured directly for this study, collected from manufacturers' specification sheets, or obtained from other publicly available databases (Supplementary Table 1). Light source emissions are typically available as spectral radiance (W sr^{-1} m^{-2} nm^{-1}). Light source intensity was first scaled so that the reflected radiance from a white (100% reflectance) surface being illuminated below an artificial light matched human-specific luminance values of 10, 1, 0.1, 0.01 and 0.001 cd.m^{-2}. These intensities cover the range typical of artificial light at night, from a surface suspended directly below the bulb (10 cd.m^{-2}), to surfaces a few metres away (1 - 0.01 range), and low-level skyglow similar to starlight (0.001). We performed this step because any artificial light is primarily intended for illumination for human viewers, so each was scaled for equal human-specific viewing intensities. We used the standard CIE photometric luminosity function to calculate the luminance of each source:

$$\Phi v = 683 \int_{\lambda=300\,nm}^{\lambda=700\,nm} \Phi e_{\lambda} Y_{\lambda}$$

(Supplementary Equation 1)

Φ_v is the luminous flux in lumens (cd m^{-2}), $\Phi_{e,\lambda}$ is the spectral radiant flux (W sr^{-1} m^{-2} nm^{-1}), Y_λ is the luminosity function based on the CIE 2006 2 degree observer Y sensitivity curve (we chose to use the photopic y channel rather than rod-based curve because artificial light is generally intended to recreate photopic/mesopic light levels sufficient for cone-based colour vision in humans), λ is the wavelength in nanometres. This and all other calculations were performed at wavelength increments of 1nm from 300 to 700nm.

Following calculation of luminous flux, the reflected radiance scaled to a given intensity (*Ls*, W sr^{-1} m^{-2} nm^{-1}) was calculated as:

$$Ls_{\lambda} = \Phi e_{\lambda} R_{\lambda} \frac{T}{\Phi v_{\lambda}}$$

(Supplementary Equation 2)

Where T is the target luminance (e.g. 10 cd.m^{-2}).

Spectral radiance was then converted into photon flux *Lp* (photons s^{-1} sr^{-1} $microns^{-2}$ nm^{-1}):

$$Lp_\lambda = 5{,}030\, Ls_\lambda\, \lambda$$

(Supplementary Equation 3)

Hawkmoth absolute quantum catch values *N* were then calculated using a modification of Johnsen et al. (2006):

$$N_i = 1.13 \left(\frac{\pi}{4}\right) n\, \Delta\rho^2 D^2 \Delta t \int_{\lambda=300\,nm}^{\lambda=700\,nm} \kappa\tau \left(1 - e^{-kSi_\lambda l}\right) Lp_\lambda$$

(Supplementary Equation 4)

Where S specifies the sensitivity functions of the elephant hawkmoth's (*Deilephila elpenor*) three receptor types *i*; MW, SW and UV (provided by S. Johnsen). The remaining parameter descriptions and values are shown in Supplementary Table 4.

<u>Supplementary Table 4:</u> Parameters used for modelling hawkmoth cone catch quanta. Values taken from Johnsen et al. Table 1[1], with the exception of integration time, which was taken from Stöckl & Kelber[2].

**Parameter**	**Description**	**Value**
n	Effective facets in the superposition aperture	568
Δρ	Photoreceptor acceptance angle	0.0524 radians
D	Diameter of facet lens	29 µm
κ	Quantum transduction efficiency	0.5
τ	Fractional transmission of ocular media	0.8
Δt	Integration time of photoreceptor	1 s

k	Absorption coefficient of the rhabdom	0.0067 µm
l	Rhabdom length (doubled by tapetal reflection)	414 µm

Blue tit absolute quantum catch values *N* were calculated similarly, by modifying the method used by Olsson et al.[3]. We adapted this model (based on chicken vision) to use blue tit eye morphology (i.e. blue tit pupil diameter from Lind, Kelber & Kroger[4]) and spectral sensitivity curves[5]:

$$N_i = \left(\frac{\pi}{4}\right)^2 \left(\frac{d}{f}\right)^2 D^2 \kappa \tau \Delta t \int_{\lambda=300\,nm}^{\lambda=700\,nm} \left(1 - e^{-k Si_\lambda \Omega_i l}\right) Lp_\lambda$$

(Supplementary Equation 5)

Where S specifies the sensitivity functions of the bluetit's (*Cyanistes caeruleus*) four receptor types *i* LW, MW, SW and UV[5], and Ω applies a correction of the filtering by the oil droplets (specific to each receptor type). The parameter descriptions and values are shown in Supplementary Table 5.

Supplementary Table 5: Parameters used for modelling blue tit cone catch quanta.

**Parameter**	**Description**	**Value**
l	Cone outer segment length	30 µm
k	Absorption coefficient	0.035
d	Ellipsoid diameter	3.1 µm
f	Focal length	5187.5 µm
D	Pupil diameter	2000 µm
κ	Quantum transduction efficiency	0.5

τ	Fractional transmission of ocular media	0.8
Δt	Integration time of photoreceptor	0.025, 0.05, 0.05 s for intensities of 10, 1 & 0.1 cd.m^{-2} resp.
Ω	Oil droplet transmission (LW, MW, SW, UV)	0.5, 0.5, 0.6, 0.8

Coordinates in RNL space, and colour contrast

Absolute quantal catch values were then used to calculate XY (trichromatic; hawkmoth) or XYZ (tetrachromatic; blue tit) coordinates in the Receptor Noise Limited (RNL) colour space[6-9]. We assumed the most abundant cone-class had a conservative noise-to-signal ratio of 0.05 in all cases (note that we re-ran the analysis with a ratio of 0.1, and this did not qualitatively change any findings, see analysis code), and adjusted receptor-specific Weber fractions ω_i based on cone ratios, which were assumed to be 1:1:7 for the hawkmoth[10], and 1: 1.92: 2.68: 2.7 for blue tit[5]. Receptor-specific Weber fractions were then modified following the photon shot noise model e_i[3].

$$e_i = \frac{\sqrt{\omega_i^2 N_i^2 + N_i}}{N_i}$$

(Supplementary Equation 6)

where N_i is the absolute quantal catch for the receptor of type i. Note that Olsson et al.[3] calculate Ni in this equation as the average of two stimuli in a pairwise comparison, however for this study we calculate RNL colour space coordinates based on each sample (so that we can calculate volumes and distributions, not just perform pairwise colour comparisons), so we do not average between samples.

Photoreceptors tend to fire in proportion to the logarithm of photon flux, so we used the log form of the RNL model where (based on[9]):

$$n_i = \ln(Ni)$$

(Supplementary Equation 7)

RNL XYZ coordinates are then calculated using the following equations[7,9]:

$$X = \sqrt{\frac{1}{e_3^2 + e_4^2}}\left(n_4 - n_3\right)$$

(Supplementary Equation 8)

$$Y = \sqrt{\frac{e_3^2 + e_4^2}{\left(e_2 e_3\right)^2 + \left(e_2 e_4\right)^2 + \left(e_3 e_4\right)^2}}\left(n_2 - \left(n_4 \frac{e_3^2}{e_3^2 + e_4^2} + n_3 \frac{e_4^2}{e_3^2 + e_4^2}\right)\right)$$

(Supplementary Equation 9)

$$Z = A\left(n_1 - \left(a n_4 + b n_3 + c n_2\right)\right)$$

(Supplementary Equation 10)

$$A = \sqrt{\frac{\left(e_3 e_4\right)^2 + \left(e_2 e_4\right)^2 \left(e_2 e_3\right)^2}{\left(e_2 e_3 e_4\right)^2 + \left(e_1 e_3 e_4\right)^2 + \left(e_1 e_2 e_4\right)^2 + \left(e_1 e_2 e_3\right)^2}}$$

(Supplementary Equation 11)

$$a = \frac{\left(e_2 e_3\right)^2}{\left(e_3 e_4\right)^2 + \left(e_2 e_3\right)^2 + \left(e_2 e_4\right)^2}$$

(Supplementary Equation 12)

$$b = \frac{\left(e_2 e_4\right)^2}{\left(e_3 e_4\right)^2 + \left(e_2 e_3\right)^2 + \left(e_2 e_4\right)^2}$$

(Supplementary Equation 13)

$$c = \frac{\left(e_3 e_4\right)^2}{\left(e_3 e_4\right)^2 + \left(e_2 e_3\right)^2 + \left(e_2 e_4\right)^2}$$

(Supplementary Equation 14)

The above process was used to calculate the XY (hawkmoth) and XYZ (blue tit) coordinates of each reflectance sample (including flowers, petals, backgrounds and wing markings) under all different illuminant spectra, each at a range of simulated intensities (10 to 0.001 cd.m^{-2}). Chromatic contrasts between pairs of colours were then calculated as the Euclidean distance between colour coordinates in the RNL space. The R code used to perform these calculations and all of the reflectance data are provided as supplementary material.

SUPPLEMENTARY NOTES

Validation of discrimination thresholds with existing behavioural experiments

1. Elephant hawkmoth, *D. elpenor*

Experiments in Balkenius & Kelber[11] show that *D. elpenor* can make reliable choices between given green and blue stimuli under lights with an intensity of 0.01 cd.m^{-2}. We extracted reflectance spectra for green and blue stimuli from Figure 1C using WebPlotDigitizer[12] (Supplementary Fig. 11). The chromatic contrast between blue and green stimuli was then calculated using the light sources and visual modelling code developed for this study.

Results suggest the stimuli are indeed discriminable under all our tested light types (except the narrow-band low-pressure sodium lamp), at intensities of 0.01 cd.m^{-2} and lower (Supplementary Fig. 12), so our modelling is in line with behavioural evidence.

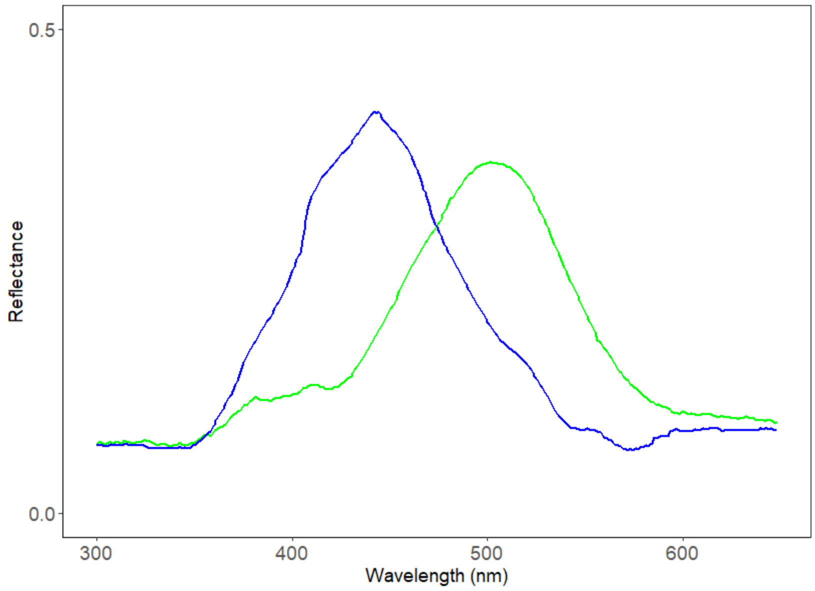

Supplementary Figure 11: Reflectance of blue and green stimuli, as extracted from Balkenius & Kelber[11], Figure 1C.

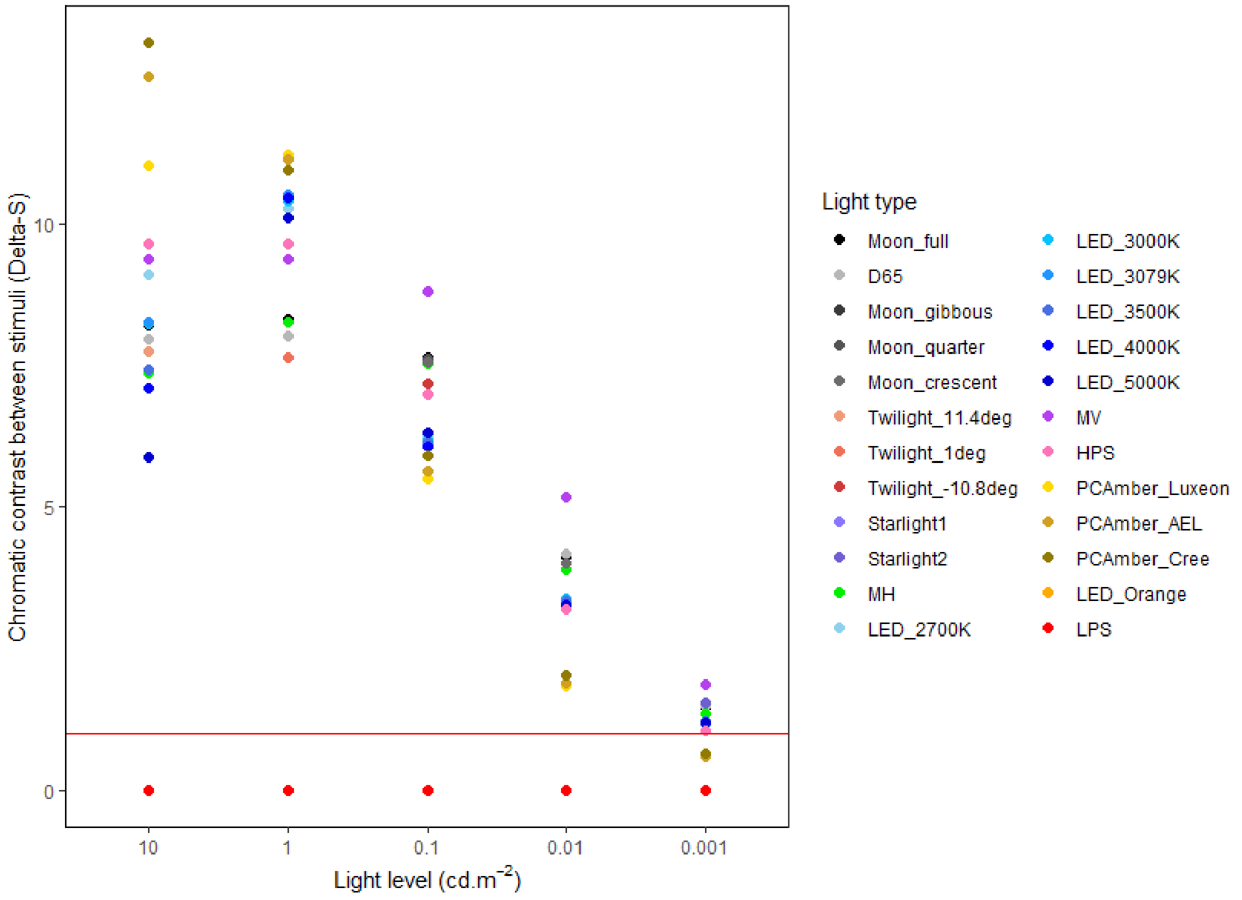

Supplementary Figure 12: Chromatic contrast between green and blue stimuli under light types and light levels used in this study. The red line indicates the putative threshold for discrimination (Delta-S = 1).

2. Blue tit *Cyanistes caeruleus*

Gomez et al.[13] tested the intensity threshold at which 3 *C. caeruleus* individuals could use colour vision to discriminate between green and blue cards. They found a threshold of 0.05 to 0.2 cd.m^{-2} for correct discrimination, depending on the individual.

Reflectance spectra for green and blue stimuli (based on cards matched for double cones) and the relative emission spectrum of the LED light used for testing were acquired from Gomez et al.[13], Figure 2, with WebPlotDigitizer[12] (Supplementary Fig. 13). The chromatic contrast between blue and green stimuli under that light type was then calculated using our visual modelling code.

According to our modelling, under the same light type as in Gomez et al.[13], the green and blue cards would be discriminable (based on Delta-S>1) at an intensity of 1 cd.m^{-2}, but would fall just below

the threshold at 0.1 cd.m^{-2} (Supplementary Fig. 14). This is broadly consistent with the intensity thresholds established by the behavioural tests of Gomez et al.[13], though it does suggest that Delta-S =1 may be a slight overestimate of the discrimination threshold for *C. caeruleus*.

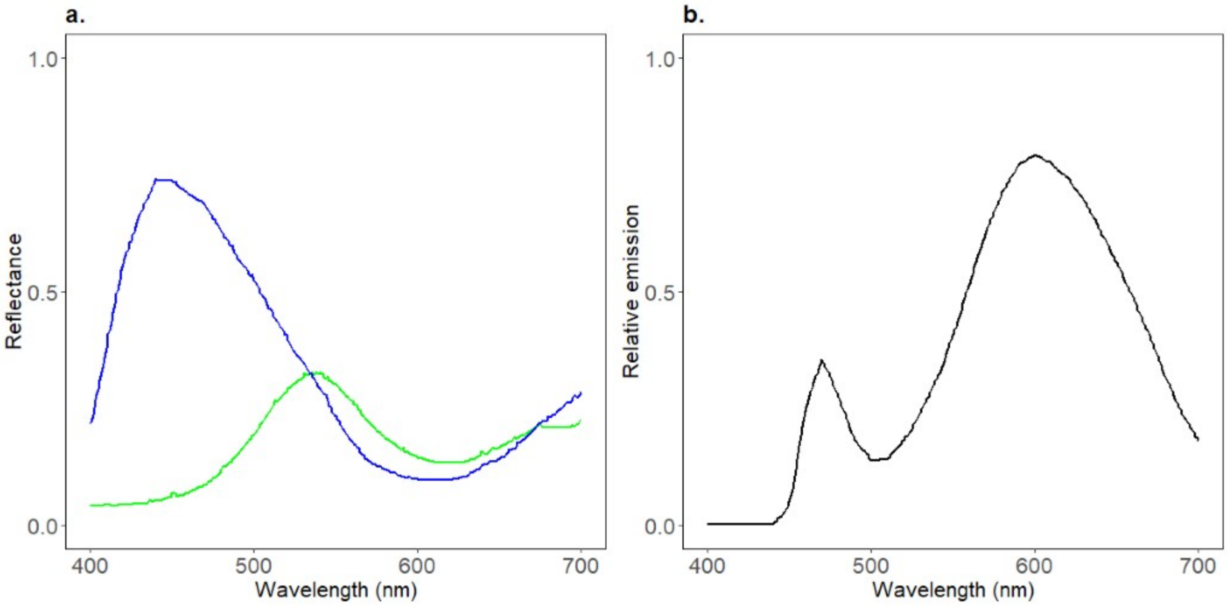

Supplementary Figure 13: Reflectance of blue and green stimuli (a) and relative emission spectrum of LED light (b), as extracted from Gomez et al.[13], Figure 2.

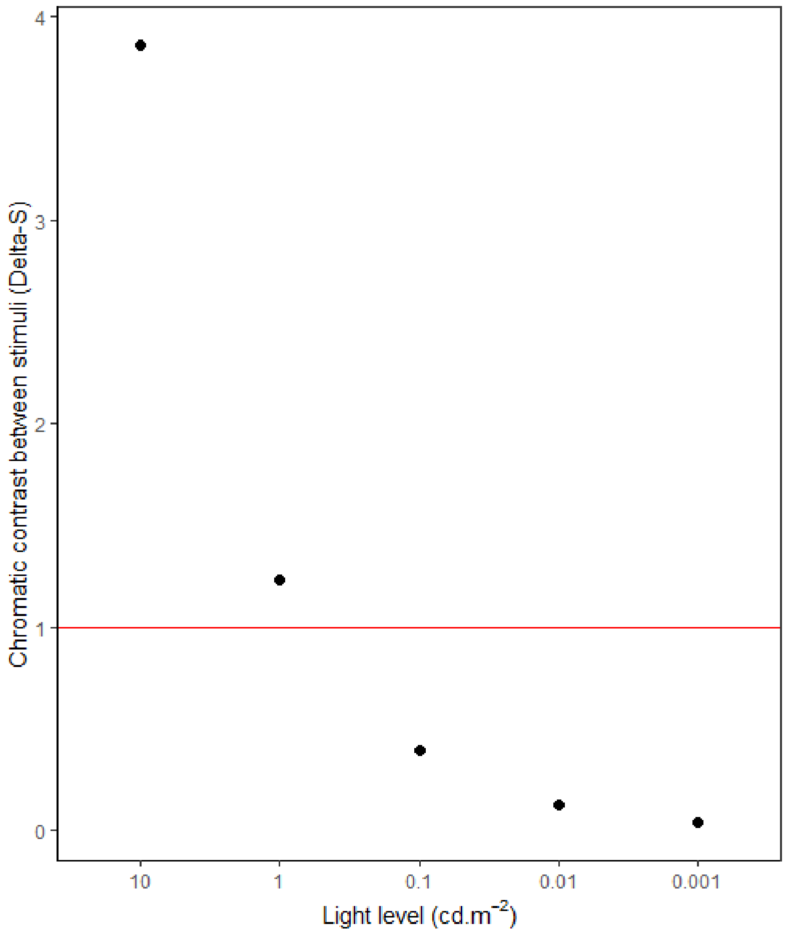

Supplementary Figure 14: Chromatic contrast between green and blue stimuli under different light levels. The red line indicates the putative threshold for discrimination (Delta-S = 1).

SUPPLEMENTARY REFERENCES

1. Johnsen, S., Kelber, A., Warrant, E., Sweeney, A. M., Widder, E. A., Lee, R. L., & Hernández-Andrés, J. Crepuscular and nocturnal illumination and its effects on color perception by the nocturnal hawkmoth *Deilephila elpenor*. *J. Exp. Biol.* **209**, 789–800 (2006).
2. Stöckl, A. L. & Kelber, A. Fuelling on the wing: sensory ecology of hawkmoth foraging. *J. Comp. Physiol. A*, **205**, 399-413 (2019).
3. Olsson, P., Lind, O., & Kelber, A. Bird colour vision: behavioural thresholds reveal receptor noise. *J. Exp. Biol.* **218**, 184–193 (2015).
4. Lind, O. E., Kelber, A., & Kröger, R. H. Multifocal optical systems and pupil dynamics in birds. *J. Exp. Biol.* **211**, 2752–2758 (2008).
5. Hart, N. S., Partridge, J. C., Cuthill, I. C., & Bennett, A. T. Visual pigments, oil droplets, ocular media and cone photoreceptor distribution in two species of passerine bird: the blue tit (*Parus caeruleus* L.) and the blackbird (*Turdus merula* L.). *J. Comp. Physiol. A* **186**, 375–387 (2000).
6. Vorobyev, M., & Osorio, D. Receptor noise as a determinant of colour thresholds. *Proc. R. Soc. B* **265**, 351–358 (1998).
7. Hempel de Ibarra, N., Giurfa M., & Vorobyev M. Discrimination of coloured patterns by honeybees through chromatic and achromatic cues. *J. Comp. Physiol. A* **188**, 503–512 (2002).
8. Kelber, A., Vorobyev, M., & Osorio, D. Animal colour vision - behavioural tests and physiological concepts. *Biol. Rev.* **78**, 81–118 (2003).

9. Renoult, J. P., Kelber, A., & Schaefer, H. M. Colour spaces in ecology and evolutionary biology. *Biol. Rev.* **92**, 292–315 (2017).
10. Kelber, A., Balkenius, A. & Warrant, E. J. Colour vision in diurnal and nocturnal hawkmoths. *Integr. Comp. Biol.* **43**, 571–579 (2003).
11. Balkenius, A. & Kelber, A. Colour constancy in diurnal and nocturnal hawkmoths, *J. Exp. Biol.* **207**, 3307-16 (2004).
12. Rohatgi, A. WebPlotDigitizer: Version 4.4. URL: https://automeris.io/WebPlotDigitizer [Accessed January 2019].
13. Gomez, D., Grégoire, A., Del Rey Granado, M., Bassoul, M., Degueldre, D., Perret, P., & Doutrelant, C. The intensity threshold of colour vision in a passerine bird, the blue tit (*Cyanistes caeruleus*). *J. Exp. Biol.* **217**, 3775–3778 (2014).
14. van den Berg, C. P., Troscianko, J., Endler, J. A., Marshall, N. J. & Cheney, K. L. Quantitative Colour Pattern Analysis (QCPA): A comprehensive framework for the analysis of colour patterns in nature. *Methods Ecol. Evol.* **11**, 316–332 (2020).
15. Longcore, T. et al. Rapid assessment of lamp spectrum to quantify ecological effects of light at night. *J. Exp. Zool. A Ecol. Integr. Physiol.* **329**, 511–521 (2018).
16. Elvidge, C. D., Keith, D. M., Tuttle, B. D. & Baugh, K. E. Spectral identification of lighting type and character. *Sensors*. **10**, 3961-3988 (2010).
17. Baker, H. G. The adaptation of flowering plants to nocturnal and crepuscular pollinators, *Q. Rev. Biol.* **36**, 64-73 (1961).
18. Tartaglia, E. S. & Handel, S. N. Nectar plant preferences of *Hemaris* (Sphingidae: Lepidoptera) on co-occurring native *Cirsium* and non-native *Centaurea* (Asteraceae) inflorescences, *J. Pollinat. Ecol.* **13**, 184-187 (2014).
19. Emmet, A. M. & Heath, J. *The moths and butterflies of Great Britain and Ireland*. Volume 9 Part I. Sphingidae - Noctuidae (Noctuinae - Hadeninae), 288 pp., Harley Books, Colchester UK (1983).
20. Waring, P. & Townsend, M. *Field guide to the moths of Great Britain and Ireland*, 444 pp., British Wildlife Publishing Ltd., Totnes UK (2009).
21. Yamada, T., Kodama, K. & Maki, M. Floral morphology and pollinator fauna characteristics of island and mainland populations of *Ligustrum ovalifolium* (Oleaceae), *Biol. J. Linn. Soc.* **174**, 489-501 (2014).
22. Ottosen, C.-O. Pollination ecology of *Lonicera periclymenum* L. in N-E.-Zealand, Denmark: floral development, nectar production & insect visits, *Flora* **178**, 271-279 (1986).
23. MacGregor, C. J., Kitson, J. J. N., Fox, R., Hahn, C., Lunt, D. H., Pocock, M. J. O. & Evans, D. M. Construction, validation, and application of nocturnal pollen transport networks in an agro-ecosystem: a comparison using light microscopy and DNA metabarcoding, *Ecol. Entomol.*, **44**, 17-29 (2019).
24. Kawaano, S., Odaki, M., Yamaoka, R., Oda-Tanabe, M., Takeuchi, M. & Kawano, N. Pollination biology of Oenothera (Onagraceae). The interplay between floral UV-absorbancy patterns and floral volatiles as signals to nocturnal insects, *Plant Species Biol.* **10**, 31-38 (1995).
25. Comba, L., Corbet, S. A., Hunt, L. & Warren, B. Flowers, nectar and insect visits: Evaluating British plant species for pollinator-friendly gardens, *Ann. Bot.* **83**, 369-383 (1999).
26. Kephart, S., Reynolds, R. J., Rutter, M. T., Fenster, C. B. & Dudash, M. R. Pollination and seed predation by moths on *Silene* and allied Caryophyllaceae: evaluating a model system to study the evolution of mutualisms, *New Phytol*. **169**, 667-680 (2006).
27. Martins, D. J. & Johnson, S. D. Interactions between hawkmoths and flowering plants in East Africa: polyphagy and evolutionary specialization in an ecological context. *Biol. J. Linn. Soc.* **110**, 199-213 (2013).
