# Supplementary material for: Artificial nighttime lighting impacts visual ecology links between flowers, pollinators and predators
Source: Nat Commun. 2021 Jul 6;12:4163. doi: 10.1038/s41467-021-24394-0 (PMC8260664; doi:10.1038/s41467-021-24394-0)
Supplement: Supplementary file 3 — Reporting Summary [file 41467_2021_24394_MOESM3_ESM.pdf]

Corresponding author(s): Jolyon Troscianko

Last updated by author(s): Jun 15, 2021

Reporting Summary

Nature Research wishes to improve the reproducibility of the work that we publish. This form provides structure for consistency and transparency in reporting. For further information on Nature Research policies, see our Editorial Policies and the Editorial Policy Checklist.

Statistics

For all statistical analyses, confirm that the following items are present in the figure legend, table legend, main text, or Methods section.

n/a	Confirmed	
☐	☒	The exact sample size (*n*) for each experimental group/condition, given as a discrete number and unit of measurement
☐	☒	A statement on whether measurements were taken from distinct samples or whether the same sample was measured repeatedly
☐	☒	The statistical test(s) used AND whether they are one- or two-sided<br>*Only common tests should be described solely by name; describe more complex techniques in the Methods section.*
☐	☒	A description of all covariates tested
☐	☒	A description of any assumptions or corrections, such as tests of normality and adjustment for multiple comparisons
☐	☒	A full description of the statistical parameters including central tendency (e.g. means) or other basic estimates (e.g. regression coefficient) AND variation (e.g. standard deviation) or associated estimates of uncertainty (e.g. confidence intervals)
☐	☒	For null hypothesis testing, the test statistic (e.g. *F*, *t*, *r*) with confidence intervals, effect sizes, degrees of freedom and *P* value noted<br>*Give P values as exact values whenever suitable.*
☒	☐	For Bayesian analysis, information on the choice of priors and Markov chain Monte Carlo settings
☒	☐	For hierarchical and complex designs, identification of the appropriate level for tests and full reporting of outcomes
☐	☒	Estimates of effect sizes (e.g. Cohen's *d*, Pearson's *r*), indicating how they were calculated

Our web collection on statistics for biologists contains articles on many of the points above.

Software and code

Policy information about availability of computer code

Data collection	No automated data collection was used. Existing libraries of data were manually collated (described in full)
Data analysis	R version 3.5.2

For manuscripts utilizing custom algorithms or software that are central to the research but not yet described in published literature, software must be made available to editors and reviewers. We strongly encourage code deposition in a community repository (e.g. GitHub). See the Nature Research guidelines for submitting code & software for further information.

Data

Policy information about availability of data

All manuscripts must include a data availability statement. This statement should provide the following information, where applicable:

- Accession codes, unique identifiers, or web links for publicly available datasets
- A list of figures that have associated raw data
- A description of any restrictions on data availability

Code and data are available to download as supplementary material

Field-specific reporting

Please select the one below that is the best fit for your research. If you are not sure, read the appropriate sections before making your selection.

☐ Life sciences ☐ Behavioural & social sciences ☒ Ecological, evolutionary & environmental sciences

For a reference copy of the document with all sections, see nature.com/documents/nr-reporting-summary-flat.pdf

Ecological, evolutionary & environmental sciences study design

All studies must disclose on these points even when the disclosure is negative.

Study description	Modeling the likely impact of artificial light on nocturnal moth visual ecology
Research sample	Samples of moth wing reflectance spectra were sourced from museums (all 14 nocturnal species found in Europe, 5 specimens of each species measured in multiple locations, giving a total of 70 individuals and 445 samples). Floral reflectance spectra were collected from fieldwork or from published databases. When collected manually, 10 samples from each species were taken. A total of 1,145 floral/leaf reflectance spectra samples. Species were selected based on published observations of feeding from hawkmoths (all listed in the manuscript).
Sampling strategy	5 independent museum specimens of moths was considered sufficient. Gathering more would have meant trapping live moths (along with ethical considerations, RRR etc.) 10 samples from each plant was considered sufficient.
Data collection	Data were collected from museum specimens, and from fieldwork. Fieldwork involved searching for target species and gathering multiple samples
Timing and spatial scale	Wildflowers were collected during their respective flowering periods in 2018.
Data exclusions	No data were excluded
Reproducibility	We have provided the code and data used for our study so that anyone can reproduce our analysis.
Randomization	Species were considered discrete in the statistical models.
Blinding	Blinding was not necessary because of the balanced design. i.e. the experimenter knowing our hypothesis would not have been able to collect samples in a way which would bias the results.

Did the study involve field work? ☒ Yes ☐ No

Field work, collection and transport

Field conditions	Primarily walking around campus and private gardens, searching for wildflowers
Location	Cornwall, UK
Access & import/export	All samples were collected with permission of the land owners.
Disturbance	A small number of petals and leaves were collected from each wildflower. None were of conservation concern. Museum specimens were unharmned

Reporting for specific materials, systems and methods

We require information from authors about some types of materials, experimental systems and methods used in many studies. Here, indicate whether each material, system or method listed is relevant to your study. If you are not sure if a list item applies to your research, read the appropriate section before selecting a response.

Materials & experimental systems

n/a	Involved in the study
☒	☐ Antibodies
☒	☐ Eukaryotic cell lines
☒	☐ Palaeontology and archaeology
☐	☒ Animals and other organisms
☒	☐ Human research participants
☒	☐ Clinical data
☒	☐ Dual use research of concern

Methods

n/a	Involved in the study
☒	☐ ChIP-seq
☒	☐ Flow cytometry
☒	☐ MRI-based neuroimaging

Animals and other organisms

Policy information about studies involving animals; ARRIVE guidelines recommended for reporting animal research

Laboratory animals	Did not involve
Wild animals	No wild animals
Field-collected samples	Plant samples were transferred to the dark room within hours of collection, and were kept watered.
Ethics oversight	Ethical approval was granted by Exeter University

Note that full information on the approval of the study protocol must also be provided in the manuscript.
